# Large-scale EM data reveals myelinated axonal changes and altered connectivity in the corpus callosum of an autism mouse model

**DOI:** 10.3389/fninf.2025.1563799

**Published:** 2025-04-11

**Authors:** Guoqiang Zhao, Ao Cheng, Jiahao Shi, Peiyao Shi, Jun Guo, Chunying Yin, Hafsh Khan, Jiachi Chen, Pengcheng Wang, Jiao Chen, Ruobing Zhang

**Affiliations:** ^1^School of Biomedical Engineering (Suzhou), Division of Life Sciences and Medicine, University of Science and Technology of China, Hefei, China; ^2^Key Laboratory of Medical Optics, Suzhou Institute of Biomedical Engineering and Technology, Chinese Academy of Sciences, Suzhou, China; ^3^School of Electronic and Information Engineering, Soochow University, Suzhou, China; ^4^Hefei Comprehensive National Science Center, Institute of Artificial Intelligence, Hefei, China

**Keywords:** Shank3b, autism spectrum disorder, corpus callosum, serial scanning electron microscopy, myelinated axons

## Abstract

**Introduction:**

Autism spectrum disorder (ASD) encompasses a diverse range of neurodevelopmental disorders with complex etiologies, including genetic, environmental, and neuroanatomical factors. While the exact mechanisms underlying ASD remain unclear, structural abnormalities in the brain offer valuable insights into its pathophysiology. The corpus callosum, the largest white matter tract in the brain, plays a crucial role in interhemispheric communication, and its structural abnormalities may contribute to ASD-related phenotypes.

**Methods:**

To investigate the ultrastructural alterations in the corpus callosum associated with ASD, we utilized serial scanning electron microscopy (sSEM) in mice. A dataset of the entire sagittal sections of the corpus callosum from wild-type and Shank3B mutant mice was acquired at 4 nm resolution, enabling precise comparisons of myelinated axon properties. Leveraging a fine-tuned EM-SAM model for automated segmentation, we quantitatively analyzed key metrics, including G-ratio, myelin thickness, and axonal density.

**Results:**

In the corpus callosum of Shank3B autism model mouse, we observed a significant increase in myelinated axon density, accompanied by thinner myelin sheaths compared to wild-type. Additionally, we identified abnormalities in the diameter distribution of myelinated axons and deviations in G-ratio. Notably, these ultrastructural alterations were widespread across the corpus callosum, suggesting a global disruption of myelinated axon integrity.

**Discussion:**

This study provides novel insights into the microstructural abnormalities of the corpus callosum in ASD mouse, supporting the hypothesis that myelination deficits contribute to ASD-related communication impairments between brain hemispheres. However, given the structural focus of this study, further research integrating functional assessments is necessary to establish a direct link between these morphological changes and ASD-related neural dysfunction.

## 1 Introduction

Autism Spectrum Disorder (ASD) represents multifaceted neurodevelopmental conditions, primarily characterized by deficits in social communication and repetitive behavior patterns (Lord et al., 2020). Over the past several decades, the understanding of ASD has evolved, emphasizing the need for deeper exploration into its structural and functional underpinnings (Lord et al., 2018). Recent advances in genomics and neuroscience have identified numerous genes associated with ASD (Ellegood et al., 2015; Lim et al., 2022; Sebat et al., 2007; Zhou et al., 2019), among which Shank3 stands out for its pivotal role in synapse formation and functional maintenance (Boccuto et al., 2013; Moessner et al., 2007). Deletions or mutations of the Shank3 gene have been strongly implicated in ASD development, making it a focal point of research into the disorder's mechanisms (Boccuto et al., 2013; Crawley, 2012; Mei et al., 2016; Peça et al., 2011).

Researchers have extensively used Shank3B knockout and heterozygous mouse models as essential tools to study ASD (Cope et al., 2023; Liu et al., 2024; Li, 2024; Szabó et al., 2024; Balasco et al., 2022; Jiao et al., 2024). These models exhibit not only behavioral traits reminiscent of ASD but also significant brain structural abnormalities, including altered hippocampus and thalamus sizes, as well as disruptions in white matter organization, all of which contribute to impaired brain connectivity (Jesse et al., 2020; Balasco et al., 2022). Functional Magnetic Resonance Imaging (fMRI) studies in ASD patients reveal hypo- or hyperactivation of brain regions and deficits in resting-state functional connectivity, often linked to anomalies in white matter microstructure (Dekhil et al., 2020; Scott-Van Zeeland et al., 2010). Diffusion tensor imaging (DTI) studies have also shown reduced integrity in critical white matter tracts, such as the corpus callosum and the arcuate fasciculus (Hernandez et al., 2015), suggesting compromised signal transmission efficiency across brain regions.

The corpus callosum (CC), the largest white matter structure in the brain, is a vital conduit for interhemispheric communication (Paul et al., 2007), facilitating integration between cortical and subcortical tracts across various lobes (Hofer and Frahm, 2006; van der Knaap and van der Ham, 2011). The “atypical connectivity” theory (Belmonte et al., 2004; Frith, 2004) of ASD suggests that disrupted development in higher-order association areas leads to abnormal brain connectivity patterns (Catani et al., 2016). Macrostructural findings, such as reduced volume and altered development trajectories of the corpus callosum in individuals with ASD, underscore its importance in understanding the disorder (Badhe et al., 2024; Kirkovski et al., 2024).

Within the corpus callosum, myelinated axons account for 90% of its fibers (Riise and Pakkenberg, 2011), playing a crucial role in efficient neural communication through rapid signal conduction (Almeida and Lyons, 2017; Suminaite et al., 2019). Thus, Exploring the structural properties of these myelinated fibers is key to understanding the connectivity deficits observed in ASD. While previous studies using scanning electron microscopy (SEM) have provided valuable insights into the corpus callosum's ultrastructure, they primarily focused on healthy models or non-ASD conditions (Lee et al., 2019; West et al., 2015). Furthermore, these investigations often examined small, localized regions, failing to capture a comprehensive view of the corpus callosum's microstructural connectivity and its functional implications in ASD.

Emerging evidence from studies of Shank3B mouse models suggests that myelination changes may be linked to ASD symptoms (Malara et al., 2022). However, a detailed examination of how Shank3B deficiency impacts the ultrastructure of the corpus callosum remains unexplored. In this study, we leverage serial SEM and deep learning-based image analysis to create a high-resolution map of myelinated axons in the corpus callosum of Shank3B heterozygous mouse. By uncovering critical microstructural alterations, this research aims to advance the understanding of ASD-associated connectivity deficits and pave the way for future therapeutic strategies.

## 2 Materials and methods

### 2.1 Animals

The experiment mice used in this study were two non-littermate individuals, one wild-type and one Shank3B+/- mutant, both derived from the C57BL/6J strain. Animal experimentation was conducted at the Experimental Animal Center of the Suzhou Institute of Biomedical Engineering and Technology, Chinese Academy of Sciences. Animal experiments were performed in compliance with ethical standards. Mice were housed under standard temperature, lighting, and humidity conditions in ventilated chambers, with unrestricted access to food and water. All procedures adhered to the “Guidelines for Ethical Conduct in the Care and Use of Animals in Research” (Guo-Ke-Fa-Cai-Zi [2016] No. 398) and the “Code of Conduct in Laboratory Animal Management of Jiangsu Province” (promulgated by Order No. 45 of the People's Government of Jiangsu Province). The study protocol was reviewed and approved by the institutional ethics committee and carried out in full accordance with its guidelines.

### 2.2 Sample preparation

Samples were collected from two 5-week-old mice, one wild-type and one Shank3B heterozygous. Animals were fixed by cardiac perfusion with a solution containing 2.5% paraformaldehyde (PFA), 2.5% glutaraldehyde, 0.15 M cacodylate buffer and 0.05 M calcium chloride. Following perfusion, brain tissue was harvested, and initially sectioned sagittally into large blocks using a mouse brain slice mold (Beijing Jitai Yuancheng Technology, spacing 1 mm). Subsequently, a Leica VT1200S vibrating blade microtome was used to further slice the sagittal blocks at 100 μm intervals, enabling a precise approach to the exact midline plane with an accuracy of 100 μm. The mid-sagittal slice of the corpus callosum was then further fixed in the same solution, chemically stained for enhanced contrast, dehydrated with anhydrous ethanol and acetone, and embedded in epoxy resin; detailed experimental procedures can be found in previously reported protocols (Fan et al., 2021). The resin block was mounted on an ultramicrotome (Leica UC7). Sections were cut with a diamond knife at 50 nm thickness, collected on a carbon-coated Kapton tape, and restained with heavy metals. Finally, the sections were affixed to silicon wafers using conductive adhesive tape for storage and subsequent imaging.

### 2.3 Image acquisition

Sample sections were imaged using a Zeiss MultiSEM 505 at a lateral resolution of 4 nm, with the following acquisition parameters: a single-beam electron current of 570 pA, a landing voltage of 1.5 kV, a working distance of 1.4 mm, and a dwell time of 0.8 μs. This setup enabled the successful acquisition of high-resolution electron microscopy images of the entire corpus callosum sections from both wild-type and Shank3B heterozygous mouse.

### 2.4 Image processing

To reconstruct the entire section from the raw data, we utilized the overlapping regions of adjacent tiles for stitching and rendering. The large data size and high resolution imposed stringent demands on stitching accuracy while also constraining processing speed. To balance these factors, we employed a hybrid feature stitching algorithm based on error detection, previously developed by our team (Shi et al., 2024). This algorithm integrates multiple feature-based methods to optimize computational accuracy and speed, significantly improving stitching efficiency without compromising precision.

To achieve high-precision segmentation of myelinated axons and myelin sheaths, we developed EM-SAM, a deep-learning model based on the Segment Anything Model (SAM) (Kirillov et al., 2023b). SAM is a large-scale, generalizable image segmentation model designed to handle diverse image tasks. Building upon its robust feature extraction capabilities, EM-SAM integrates a ViT-based SAM backbone with our custom U-shaped decoding structure, enabling end-to-end segmentation tailored for electron microscopy data (Cheng et al., 2023). The model was trained on a manually annotated dataset, running 150,000 training iterations with a batch size of 2 on two NVIDIA 3090 GPUs, and the entire training process took 25 hours. After training completion, the model was applied to the entire dataset, with the inference process taking 30 hours. Following this, four months were spent manually refining the segmentation results to ensure accuracy. For visualizations of the model's performance evaluation, please refer to Supplementary Figure 1S. We compared the proposed model with mainstream U-Net (Ronneberger et al., 2015) segmentation models and the ViT-based UNetR (Hatamizadeh et al., 2022), using the Dice coefficient and mIoU as evaluation metrics. Detailed results are provided in Supplementary Table 1S, where EM-SAM demonstrated the best performance in both axon segmentation and myelin segmentation tasks. Using this model, we achieved highly accurate semantic segmentation of myelinated axons. To obtain precise instance labels, we further processed the segmentation results using traditional image processing methods, such as connected-component labeling, dilation, and erosion, to ensure accurate identification of individual myelinated axons across the entire dataset.

### 2.5 Analysis

Leveraging the instance label results from full-image segmentation, we extracted key quantification metrics to characterize the structural properties of myelinated axons, converting image data into quantitative insights. All calculations were performed on patches of 2,048 × 2,048 pixels at an 8 nm resolution. The quantification metrics included.

#### 2.5.1 Axon and myelin area

The total area of axons and surrounding myelin was directly extracted from the corrected segmentation labels. This metric facilitates a comparative analysis of the mouse corpus callosum microstructures at the neuronal level.

#### 2.5.2 G-ratio

The G-ratio is a well-established and critical metric for quantifying myelinated axons, representing the degree of myelination relative to the axonal cross-sectional size. This metric is important as it is strongly associated with the conduction velocity of neuronal signals, directly affecting the efficiency of neural communication and brain function (West et al., 2016). Conventionally, the G-ratio is defined as the ratio of the inner radius (representing the axon) to the outer radius (including the myelin sheath), assuming a simplified circular axonal cross-section. However, high-resolution electron microscopy images reveal that most axonal cross-sections are irregular and non-circular, introducting significant inaccuracies in diameter-based measurements. Such geometric deviations can undermine the reliability of the G-ratio in assessing myelination properties. To address this issue, we utilized our high-precision segmentation results and adopted an area-based calculation approach,the equation is defined as:


(1) $$\begin{array}{l} G-ratio=\frac{r_{inner}}{R_{outer}}=\sqrt{\frac{\text{Axon area}}{\text{Myelin area}+\text{Axon area}}} \end{array}$$

By calculating the axonal area and the total area enclosed by the myelin sheath, and then taking the square root of the area ratio, this method minimizes measurement errors and improves the G-ratio accuracy for axons with irregular shapes.

#### 2.5.3 Axon diameter and myelin thickness

Axon diameter is a critical determinant of signal conduction speed, with larger diameters facilitating faster signal transmission by reducing internal resistance. However, this advantage comes at the cost of increased metabolic demand, necessitating a balance between conduction efficiency and energy consumption. The myelin sheath, a multilayered structure of lipids and proteins surrounding the axon, enhances conduction efficiency by providing insulation and minimizing electrical leakage. For axons with a roughly circular cross-section, diameter measurements are straightforward. However, irregularly shaped axons necessitate alternative estimation methods, such as equivalent circular diameter, fitted ellipse, or minimum enclosing circle. In this study, we adopted the fitted ellipse method, using its major and minor axes for precise diameter estimation (West et al., 2016). Accurately measuring myelin thickness across large corpus callosum sections presents additional challenges, as manual quantification is infeasible at this scale. To address this, we leveraged instance segmentation labels for all myelin sheaths and applied both fitted ellipse and distance transformation methods to automatically quantify myelin thickness with high accuracy (Lee et al., 2019). To further validate the accuracy of our quantification method, a subset of axons and myelin was manually quantified and compared with automated results, as shown in Supplementary Figure 2S. The analysis showed a margin of error within 0.05 microns.

#### 2.5.4 Density

The density of myelinated axons, defined as the number of myelinated axons per unit area, reflects their spatial distribution within a specific region and plays a crucial role in neural signal conduction, functional connectivity, and energy consumption. For example, in the white matter of the spinal cord and the brain, dense myelinated fibers facilitate rapid signal transmission between the brain and the peripheral regions. In this study, we quantified axonal density across the entire midsagittal section of mouse corpus callosum by counting the number of myelinated axons within each label patch, with each patch covering an area of 268μm^2^.

Statistical analyses were performed using R (version 4.2.0) (R Core Team, 2022). The Shapiro-Wilk test confirmed that the data followed non-normal distribution, prompting the use of the Mann-Whitney U test for comparisons. Data visualization was carried out using ggplot2 (Wickham, 2016), with detailed methods outlined in the main text and figure legends.

## 3 Results

**Figure 1:**
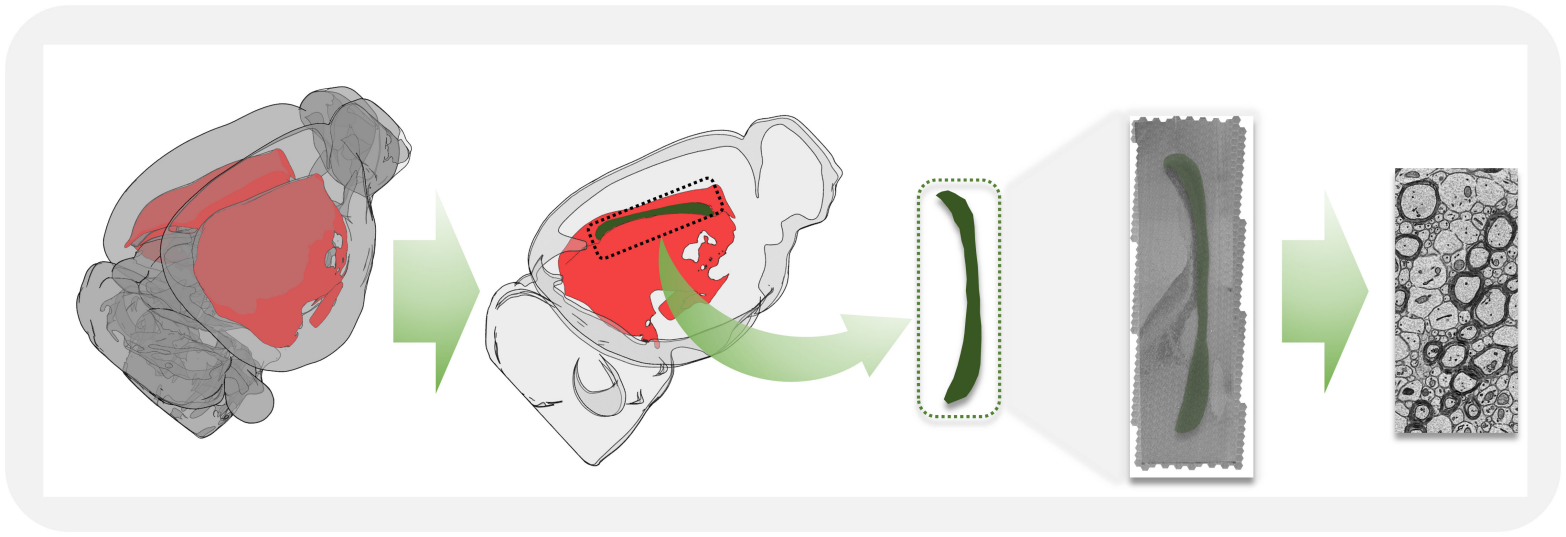
A schematic illustration showing the workflow for acquiring high-resolution electron microscopy images in the mid-sagittal plane from corpus callosum tissue.

The corpus callosum primarily mediates the information transfer and integration between the left and right cerebral hemispheres. It consists of dense white matter fibers, with projections from various cortical regions converging at the midline sagittal plane (Figure 1). For this study, we selected the mid-region of the corpus callosum, highlighted in green in Figure 1, where its morphology is most clearly observable. High-resolution imaging was performed on the two-dimensional sections, and the acquired raw image tiles were stitched into a large electron microscopy image of the entire corpus callosum midline sagittal section. Using advanced deep learning algorithms, we achieved precise segmentation of myelinated axons and myelin sheaths, enabling detailed ultrastructural analysis.

### 3.1 High-resolution corpus callosum EM dataset

**Figure 2:**
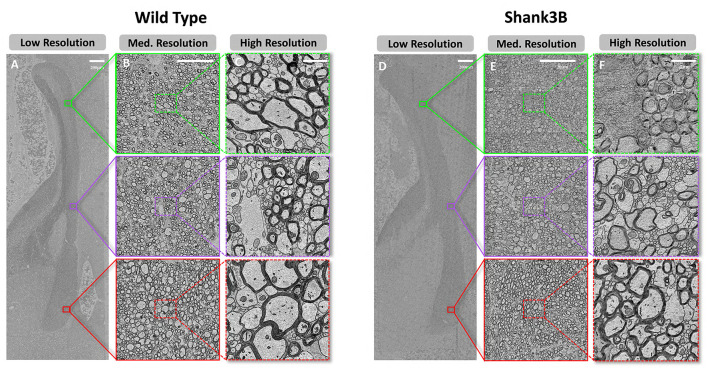
Demonstration of the dataset at multiple scales of resolution. **(A–C)**, Scanning electron microscopy images of wild-type mouse at resolution scales of low resolution(512 nm), medium resolution(64 nm), and high resulotion(8 nm) (from left to right). **(D–F)**, Scanning electron microscopy images of Shank3B mouse at different resolution scales, in the same order as wild-type.

To investigate the ultrastructural features of the corpus callosum, we constructed a high-resolution SEM dataset of the midline sagittal cross-section of corpus callosum from both a wild-type and a Shank3B heterozygous mouse (Figure 2). The dataset encompasses the entire cross-section, including the genu, body, and splenium of the corpus callosum. It comprises 59,292 tiles for the wild-type mouse and 50,447 tiles for the Shank3B heterozygous mouse. Raw image tiles were acquired at a pixel resolution of 4 nm. To enhance data processing and storage efficiency without compromising segmentation accuracy, we downsampled the images to 8 nm per pixel. The rendered dataset was divided into 4,096 × 4,096-pixel patches for processing. For the wild-type mouse, 54 patches were generated in the X direction and 165 in the Y direction, resulting in approximately 8,900 patches. For the Shank3B heterozygous mouse, 56 patches in the X direction and 139 patches in the Y direction were obtained, yielding about 7,700 patches.

To enable detailed analysis and support diverse processing requirements, we further performed gradient downsampling, producing image data at eight different resolutions: 8 nm, 16 nm, 32 nm, 64 nm, 128 nm, 256 nm, 512 nm, and 1024 nm. This multi-resolution dataset, totaling approximately 300 GB, provides flexibility for examining structures at varying scales. Figures 2A, D present an overview of the corpus callosum at 512 nm resolution, while Figures 2B, E zoom into subregions at 64 nm resolution. Figures 2C, F illustrate the finest resolution at 8 nm, clearly revealing the ultrastrctures of myelinated axons, myelin sheaths, and unmyelinated axons. These dense white matter fibers, characteristic of the corpus callosum, can be observed in most regions, forming the foundation for subsequent segmentation and analysis.

### 3.2 Automated high-precision segmentation of myelinated axons

Extracting detailed ultrastructural information from electron microscopy (EM) images has traditionally been a labor-intensive process, reliant on manual annotation by experts. While effective for small datasets, this approach is impractical for large-scale data like our corpus callosum dataset. Recent advances in deep learning algorithms, however, have paved the way for automated annotation techniques, significantly improving efficiency and scalability. Given the extensive volume of our dataset, which covers the entire midline sagittal section of the corpus callosum, we adopted the EM-SAM model (Cheng et al., 2023), a fine-tuned version of the Segment Anything Model (SAM) (Kirillov et al., 2023a), to perform high-precision segmentation of myelinated axons. This approach allowed us to achieve automated annotation across the dataset, addressing the impracticality of manual methods.

**Figure 3:**
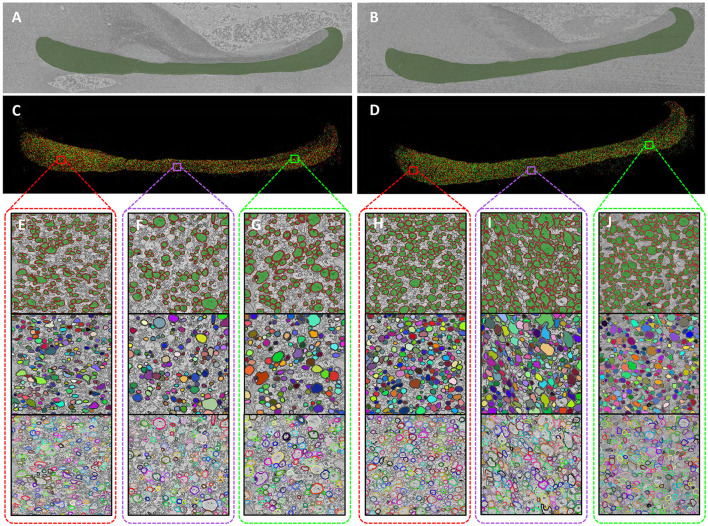
High-precision segmentation results of myelinated axons. **(A, B)**, Corpus callosum region in the sections [**(A)** Wild-type, **(B)** Shank3B]. **(C, D)**, The myelinated axon segmentation labels for the entire corpus callosum [**(C)** Wild-type, **(D)** Shank3B]. **(E–G)**, Detailed segmentation labels of different subregions in the wild-type mouse [**(E)** genu, **(F)** body, **(G)** splenium]. **(H–J)**, Detailed segmentation labels of different subregions in the Shank3B mouse [**(H)** genu, **(I)** body, **(J)** splenium].

The segmentation results are summarized in Figure 3. While the raw EM sections included portions of adjacent structures such as the Dorsal Commissure of the Fornix (DCF) and the Hippocampus (HC), these regions were excluded from further analysis. Figures 3A, B illustrate the masks highlighting the specific corpus callosum regions. Semantic segmentation results for myelinated axons within the corpus callosum are shown in Figures 3C, D. To ensure accuracy, manual corrections were applied to the model outputs. Each myelinated axon was distinctly segmented, allowing for the application of traditional image processing techniques such as connectivity labeling, dilation, and erosion to transform the semantic segmentation results into instance segmentation results. Figures 3E–J provide detailed visualizations of our segmentation outcomes, showcasing data from the genu, body, and splenium regions. For each subregion, from top to bottom, rows display semantic segmentation labels, axon instance labels, and myelin instance labels. This high-precision segmentation framework enables a comprehensive analysis of myelinated axons and their associated myelin sheaths, offering a level of detail that surpasses traditional macroscopic neuroimaging methods. By leveraging this automated approach, we have established a robust foundation for investigating axonal-level structural differences in the corpus callosum.

### 3.3 Quantitative analysis of myelinated axon ultrastructure

**Figure 4:**
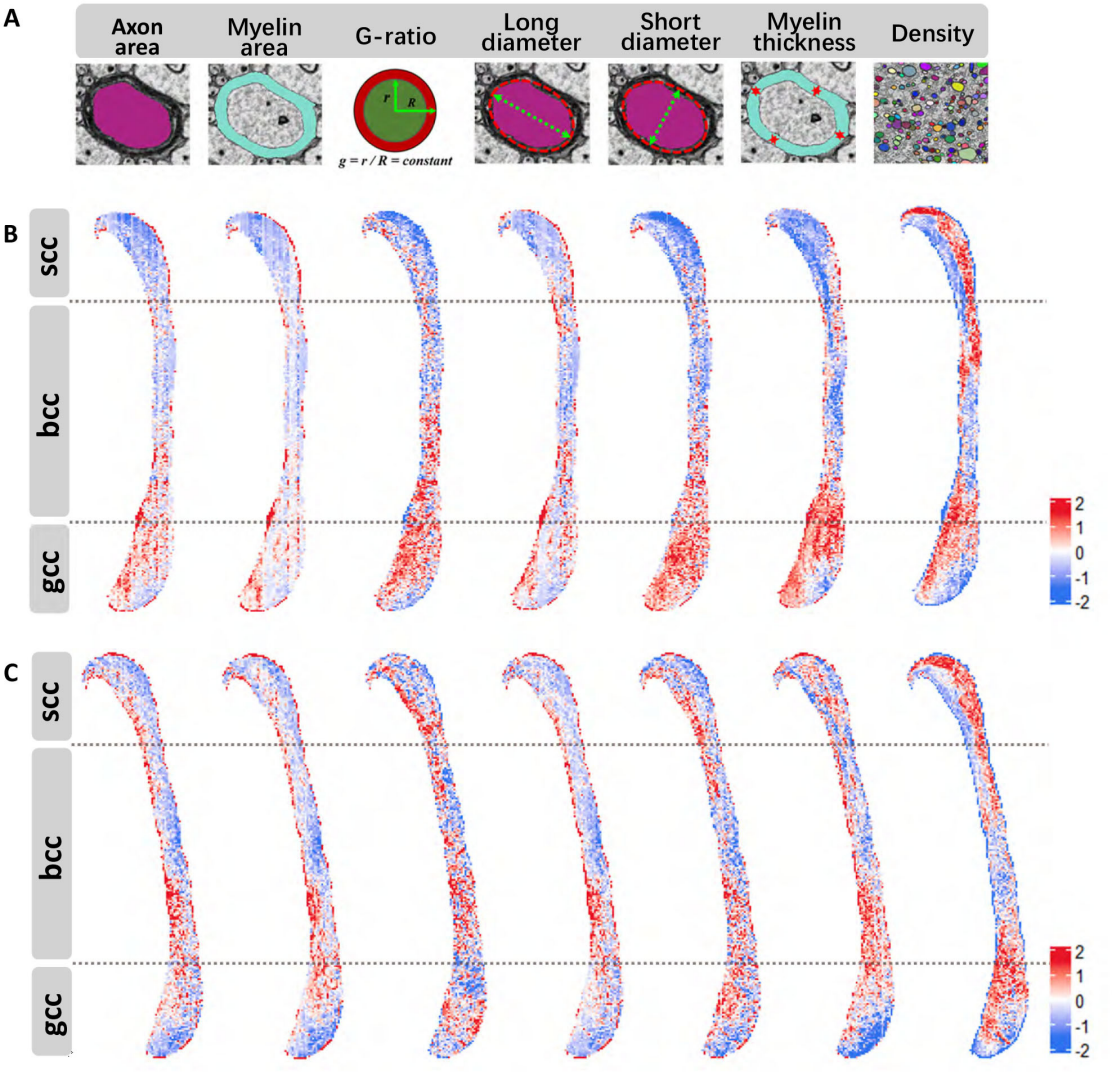
Quantitative metric results (all data standardized using z-score normalization). **(A)** Schematic representation of the calculated metrics. **(B)** Variation of various metrics across distinct regions of the corpus callosum in wild-type mouse [left-to-right order matches **(A)**]. **(C)**, Variations of different metrics in different regions of the corpus callosum in Shank3B mouse [left-to-right order matches **(A)**].

Using the instance segmentation labels generated for all myelinated axons in the midline sagittal section of the corpus callosum, we performed a comprehensive quantitative analysis of key metrics. These include axon area, myelin area, G-ratio, long and short axon diameters, myelin thickness, and density, as illustrated in Figure 4A.

Due to the irregular morphology of axons we employed an ellipse-fitting approach to calculate the long and short diameters for more accurate and consistent quantification. The computed results were specially mapped back onto the original regions in the corpus callosum, as shown in Figures 4B, C. These figures provide a visual representation of the metrics distributions across different subregions in the wild-type and Shank3B mouse corpus callosum, respectively. Our analysis revealed that the distribution patterns for several metrics were consistent across the corpus callosum in both groups. For example, axon density exhibits sparse inner regions and denser outer regions, particularly in the body and splenium, while the genu region shows a reversed trend with denser inner regions. Myelin thickness demonstrats similar spatial variation. These observations suggest that structural differences in the corpus callosum may be influenced by projections originating from different brain regions. This quantitative assessment provides a nuanced understanding of the microstructural organization of the corpus callosum and highlights the potential for structural variations to impact its functional connectivity.

### 3.4 Structural alterations in corpus callosum myelinated axons of Shank3B mouse

**Figure 5:**
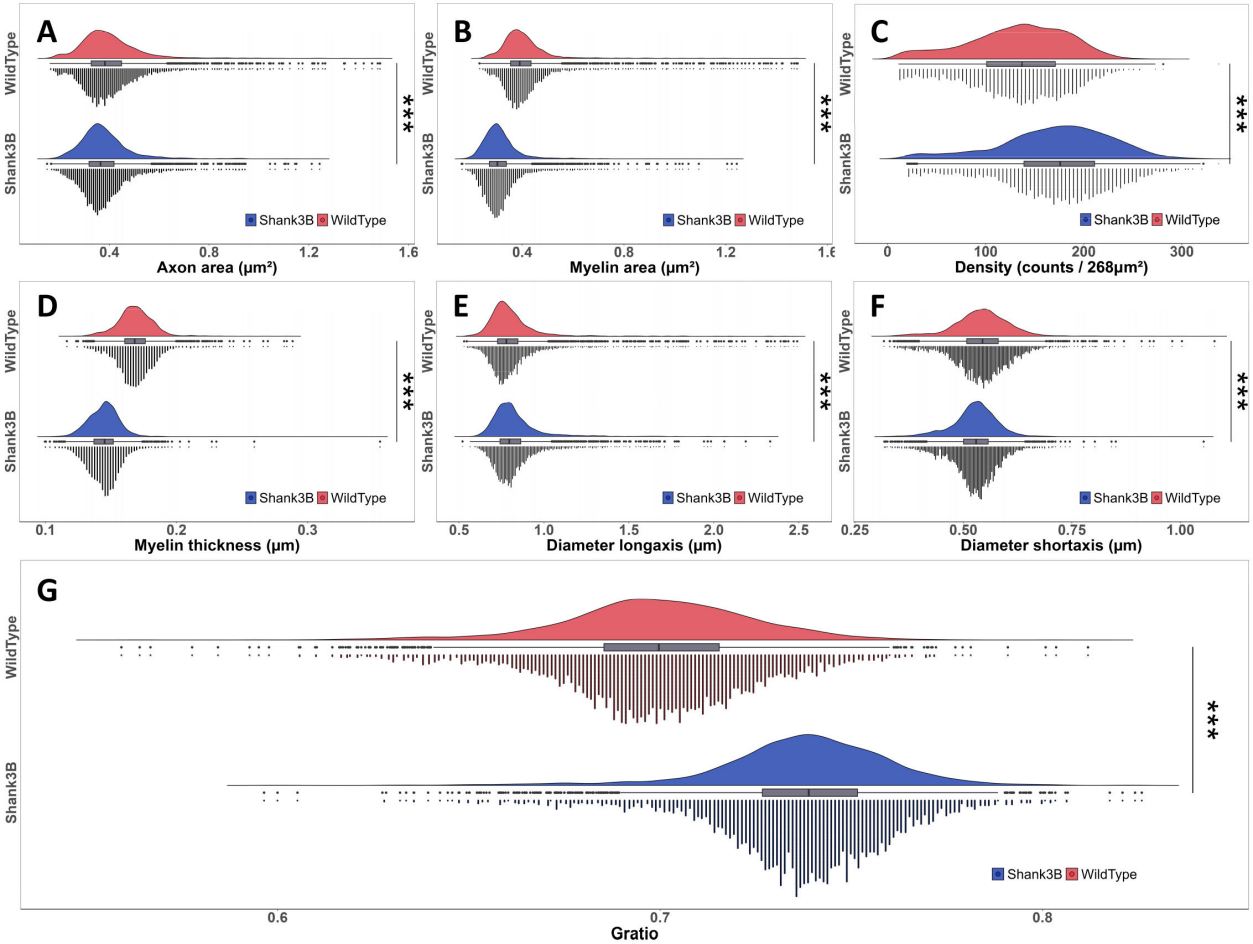
Frequency distribution histograms and statistical test results of various metrics between wild-type and Shank3B mouse (Wilcoxon rank-sum test, ***p-value < 0.001). **(A)** Axon area. **(B)** Myelin area. **(C)** Density. **(D)** Myelin thickness. **(E)** Diameter long-axis. **(F)** Diameter short-axis. **(G)** Gratio.

To further investigate the structural differences observed in the corpus callosum, we conducted a detailed statistical analysis of the extracted metrics, comparing the wild-type and Shank3B mouse. Frequency distribution histograms for each metric, including axon area, myelin area, G-ratio, myelin thickness, and axon diameters, are presented in Figures 5A–G. Quantitative results revealed distinct variations between the two groups. In the wild-type mouse, the mean ± SD for key metrics are as follows: axon area: 0.401 ± 0.135, myelin area: 0.414 ± 0.128, G-ratio: 0.699 ± 0.025, myelin thickness: 0.168 ± 0.014, long-axis diameter: 0.820 ± 0.198, and short-axis diameter: 0.543 ± 0.065. In contrast, the Shank3B mouse exhibits reduced values for axon area (0.380 ± 0.104), myelin area (0.315 ± 0.082), and myelin thickness (0.145 ± 0.012), while the G-ratio (0.738 ± 0.023) is notably elevated, indicating thinner myelin relative to axon size and suggesting the potential alterations about ultrastructural characteristics of myelination.

To assess statistical significance, we applied the Wilcoxon rank-sum test, a non-parametric method, which confirmed significant differences across all metrics (p-value < 0.001, Figures 5A–G). Particularly, the Shank3B mouse demonstrated a higher G-ratio median, consistent with compromised myelin structure, and exhibited smaller axonal cross-sections and thinner myelin sheaths compared to the wild-type mouse. While the long-axis diameters of axons remained relatively similar between the two groups, the Shank3B mouse had significantly smaller short-axis diameters, reflecting a reduction in overall axonal size. These findings suggest that the Shank3B mouse display substantial alterations in the structure of myelinated axons within the corpus callosum, which may impair neural transmission efficiency. Such structural deficits could disrupt the integration and transfer of information between hemispheres, contributing to the connectivity abnormalities associated with ASD.

Notably, segmentation results identified 625,754 myelinated axons in the Shank3B mouse, a 36.4% increase compared to the 458,671 observed in the wild-type mouse, indicating a higher axonal density in the former (Figure 5C). The elevated axon density and total axon count in the Shank3B mouse may counterbalance the transmission inefficiencies caused by structural alterations such as reduced myelin thickness, smaller axon area, and larger G-ratio—factors typically associated with diminished conduction rate. While we cannot confirm whether this results from a compensatory mechanism, this phenomenon intriguingly aligns with clinical studies that have reported increased white matter density, including in the corpus callosum, in individuals under 15 years of age with hyper-connectivity (Ecker et al., 2010; Galvez-Contreras et al., 2020). In such cases, the abnormal increase in axon numbers may lead to redundancy or confusion in signal transmission, potentially disrupting normal neural circuit function (Khanbabaei et al., 2019; Solso et al., 2016). These findings suggest a complex interplay in the Shank3B mouse between reduced single-axon efficiency and compensatory hyper-connectivity, highlighting the potential multifaceted impact of altered axonal structures on neural transmission and connectivity dynamics.

### 3.5 Global structural alterations in corpus callosum subregions

**Figure 6:**
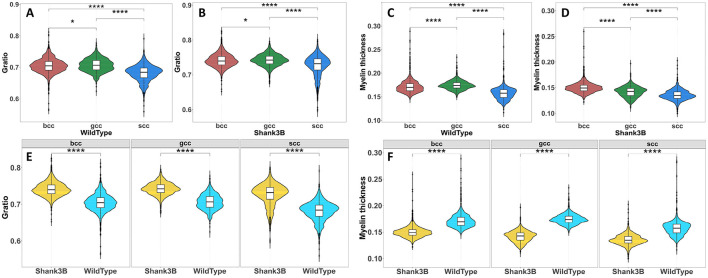
Statistical test of Gratio and myelin thickness between different subregions of the corpus callosum(Wilcoxon rank sum test, ****P < 0.001). **(A, B)**, Difference test results of Gratio in different subregions of the corpus callosum. **(C, D)**, Difference test results of myelin thickness in different subregions of the corpus callosum. **(E)**, Difference test results of Gratio in the same subregion of wild-type and Shank3B mouse. **(F)**. Difference test results of myelin thickness in the same subregion of wild-type and Shank3B mouse.

The corpus callosum is anatomically divided into distinct subregions–genu, body, and splenium–based on their cortical projections and functional characteristics, with each subregion contributing uniquely to neural circuit integration and information processing. In this study, we analyzed the midsagittal plane of the corpus callosum to determine whether structural differences observed in the Shank3B mouse are global or localized to specific subregions. Our investigation focused on two key metrics: G-ratio and myelin thickness. Pairwise comparisons of G-ratio between the Shank3B and wild-type mouse, as illustrated in Figures 6A, B, revealed significant differences across all three subregions. This underscores the diverse yet interconnected roles these subregions play in supporting cortical communication. Similarly, myelin thickness also displayed significant differences across subregions (Figures 6C, D), suggesting widespread structural abnormalities. Further within-subregion analyses, as shown in Figures 6E, F, confirmed that these variations were consistently present across different areas of the corpus callosum.

## 4 Discussion

This study provides a detailed examination of the ultrastructural and pathological changes in the corpus callosum of the Shank3B mutant mouse. Using high-resolution serial scanning electron microscopy, we generated a dataset covering the midsagittal plane of the entire corpus callosum with precise annotations of myelinated axons. To our knowledge, this represents the first large-scale, high-resolution EM imaging dataset specifically focused on white matter regions. Most existing datasets primarily emphasize gray matter or small areas of white matter, particularly in neurodevelopmental and neurodegenerative contexts (Lucchi et al., 2011; Wei et al., 2020, 2021; Abdollahzadeh et al., 2021). Our dataset addresses this gap, serving as a critical resource for both algorithm development and neuroscience research.

In addition to advancing the application of deep learning algorithms for processing large-scale neural imaging data, our study improves the accuracy of image segmentation and quantitative analysis. By converting EM images into quantitative datasets, we focused on specific biological questions, such as the structural characteristics of myelinated axons in the corpus callosum of the Shank3B ASD mouse model. This analysis revealed significant axonal structure changes, offering new insights into the pathological mechanisms underlying autism spectrum disorder, particularly the corpus callosum's role in neural information transmission. Our findings not only provide valuable biological evidence for ASD research but also support the theory of disrupted neural connectivity, highlighting the corpus callosum's critical role in this framework.

Unlike previous studies that have primarily explored the corpus callosum in ASD using macroscopic imaging techniques such as MRI and DTI, our nanoscale approach offers unprecedented detail. While macroscopic studies often examine features like volume, morphology, or overall connectivity (Del Casale et al., 2022; Goodwill et al., 2023; Zaidi et al., 2023), they fail to reveal the microscopic structural changes within the corpus callosum, particularly those affecting myelinated axons. By employing high-resolution electron microscopy, our study bridges this gap, enabling the observation and quantification of changes in myelinated axons at the nanoscale. Metrics such as G-ratio, myelin thickness, and total axon count allow for a precise understanding of microscopic pathological changes and their impact on neural transmission efficiency.

One significant finding of our study is the global nature of structural abnormalities in the myelinated axons of the Shank3B mouse. Unlike previous microscopic studies that have mainly highlighted localized differences, our data demonstrate pervasive changes across all subregions of the corpus callosum. These alterations, such as reduced myelin thickness, maybe directly affect nerve conduction velocity and, consequently, the efficiency of information transfer between the brain hemispheres. These insights suggest that myelin abnormalities may play a critical role in the neurobiological basis of ASD, potentially influencing the cognitive and behavioral symptoms observed in affected individuals.

Despite these contributions, our study has limitations that future research should address. First, our analysis is based on 2D SEM images. While this provides valuable insights, it falls short of capturing the complex 3D structure of the corpus callosum. Full 3D reconstruction, though posing significant challenges from sample preparation to data acquisition and processing, would allow for a more comprehensive understanding of spatial relationships and structural complexities within the corpus callosum. Second, while our study focuses on structural features, integrating behavioral assessments and functional analyses can further elucidate how these microstructural changes relate to ASD-associated behaviors. Future research might incorporate *in vivo* imaging techniques to explore the functional consequences of these structural differences, providing deeper insights into how corpus callosum alterations affect cognitive and behavioral outcomes. Third, due to the technical constraints of our lab in large-scale high-resolution serial EM imaging and computational capacity, the sample size in this study was relatively limited. While the observed structural differences were statistically significant, larger sample sizes will be essential to better assess their biological relevance, thereby enhancing the generalizability of our findings. Additionally, although automated quantification methods provided efficient and reproducible analyses, inherent limitations in segmentation accuracy may introduce minor discrepancies. Continued refinement of computational approaches and validation against ground truth datasets will enhance the precision and reliability of future analyses. By addressing these limitations, future research can expand on our findings to gain deeper insights into the role of corpus callosum microstructure and connectivity in ASD.

## 5 Conclusion

In this paper, we present a comprehensive analysis of microstructural changes in the corpus callosum of the Shank3B mouse model of autism, with a focus on the properties of myelinated axons. Using high-resolution scanning electron microscopy combined with deep learning-based image segmentation techniques, we quantified critical metrics such as axon area, myelin area, G-ratio, myelin thickness and axon diameter. Our results reveal significant structural differences between the Shank3B and the wild-type mouse, particularly in G-ratio, myelin thickness and axon diameter. These findings underscore the potential role of impaired myelination in the neural connectivity deficits associated with autism spectrum disorder (ASD). Specifically, the oberserved alterations in myelin thickness and G-ratio likely impair the speed and fidelity of neural signal transmission between the left and right brain hemispheres, functions critical for higher cognitive processes. Importantly, these changes are not restricted to specific subregions of the corpus callosum but are pervasive across the entire corpus callosum, indicating systemic disruptions of myelination within the Shank3B mouse model. Beyond these findings, our study contributes a valuable EM dataset of corpus callosum cross-sections, addressing the scarcity of large-scale, high-resolution EM datasets of white matter regions. This dataset not only facilitates further algorithm development but also provides a critical resource for advancing our understanding of white matter pathology in ASD and related neurodevelopmental disorders.

## Data Availability

The raw data supporting the conclusions of this article will be made available by the authors, without undue reservation.
