# Supplementary material for: Large-scale EM data reveals myelinated axonal changes and altered connectivity in the corpus callosum of an autism mouse model
Source: Front Neuroinform. 2025 Apr 11;19:1563799. doi: 10.3389/fninf.2025.1563799 (PMC12021825; doi:10.3389/fninf.2025.1563799)
Supplement: Supplementary file 1 [file Data_Sheet_1.pdf]

Supplementary Material

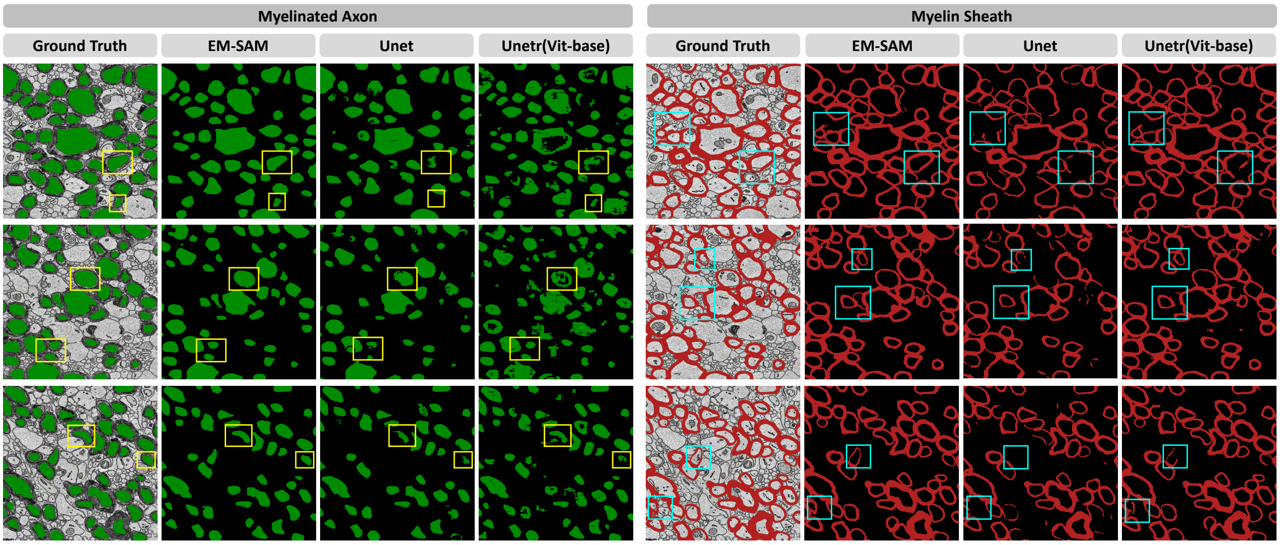

Figure S1. Compare the performance of the EM-SAM model with two widely used models, U-Net and UNetR (ViT-Base), on our dataset. Different tissue types are distinguished by colour coding: green represents axons (left) and red represents myelin sheath (right). Each row shows results for a randomly selected sample region, with three independent regions shown. Each column corresponds to the results of a specific segmentation algorithm, from left to right: EM-SAM, U-Net and UNetR (ViT-Base). Yellow and blue boxes are added to highlight regions where EM-SAM shows a significant advantage over the other two methods. All segmentation results were directly output by the models, without manual correction.

Table S1. Performance evaluation of U-Net, UNetR, and EM-SAM for axon and myelin segmentation, assessed using the Dice Similarity Coefficient and mean Intersection over Union (mIoU).

	Axon		Myelin Sheath	
**Method**	**Dice**	**mIoU**	**Dice**	**mIoU**
U-Net	0.901	0.825	0.835	0.718
UNetR (ViT-Base)	0.899	0.818	0.935	0.879
EM-SAM(ours)	**0.993**	**0.986**	**0.984**	**0.969**

**Axon Diameter**		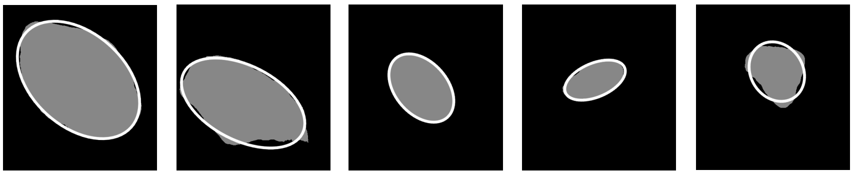				
Manual	long	2.993	2.783	1.654	1.377	1.289
	short	1.977	1.618	1.113	0.725	1.139
Our method	long	2.971	2.740	1.638	1.348	1.309
	short	1.958	1.561	1.092	0.709	1.094
**Myelin Thickness**		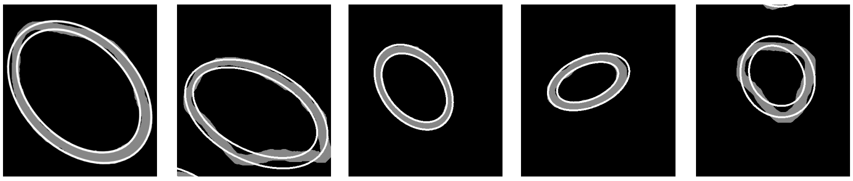				
Manual		0.212	0.211	0.166	0.196	0.178
Our method		0.202	0.197	0.152	0.191	0.207

Figure S2. Comparison of automated quantification versus manual quantification for axonal diameter and myelin thickness. Unit (μm).
